# Left Ventricular Diastolic Dysfunction Assessment with Dual-Source CT

**DOI:** 10.1371/journal.pone.0127289

**Published:** 2015-05-18

**Authors:** Zhaoying Wen, Heng Ma, Ying Zhao, Zhanming Fan, Zhaoqi Zhang, Sang Il Choi, Yeon Hyeon Choe, Jiayi Liu

**Affiliations:** 1 Department of Radiology, Beijing Anzhen Hospital, Capital Medical University, Beijing Institute of Heart, Lung and Blood Vessel Diseases, Beijing, China; 2 Department of Radiology, Yuhuangding Hospital, Yantai, Shandong Province, China; 3 Department of Echocardiology, Beijing Anzhen Hospital, Capital Medical University, Beijing Institute of Heart, Lung and Blood Vessel Diseases, Beijing, China; 4 Department of Radiology, Seoul National University Bundang Hospital, Seoul, Republic of Korea; 5 Department of Radiology and Cardiovascular and Stroke Imaging Center, Heart Vascular Stroke Institute, Samsung Medical Center, Sungkyunkwan University School of Medicine, Seoul, Korea; Sapienza University of Rome, ITALY

## Abstract

**Purpose:**

To assess the impact of left ventricular (LV) diastolic dysfunction on left atrial (LA) phasic volume and function using dual-source CT (DSCT) and to find a viable alternative prognostic parameter of CT for LV diastolic dysfunction through quantitative evaluation of LA phasic volume and function in patients with LV diastolic dysfunction.

**Materials and Methods:**

Seventy-seven patients were examined using DSCT and Doppler echocardiography on the same day. Reservoir, conduit, and contractile function of LA were evaluated by measuring LA volume (LAV) during different cardiac phases and all parameters were normalized to body surface area (BSA). Patients were divided into four groups (normal, impaired relaxation, pseudonormal, and restrictive LV diastolic filling) according to echocardiographic findings. The LA phasic volume and function in different stages of LV diastolic function was compared using one-way ANOVA analysis. The correlations between indexed volume of LA (LAVi) and diastolic function in different stages of LV were evaluated using Spearman correlation analysis.

**Results:**

LA ejection fraction (LAEF), LA contraction, reservoir, and conduit function in patients in impaired relaxation group were not different from those in the normal group, but they were lower in patients in the pseudonormal and restrictive LV diastolic dysfunction groups (*P* < 0.05). For LA conduit function, there were no significant differences between the patients in the pseudonormal group and restrictive filling group (*P* = 0.195). There was a strong correlation between the indexed maximal left atrial volume (LAVmax, r = 0.85, *P* < 0.001), minimal left atrial volume (LAVmin, r = 0.91, *P* < 0.001), left atrial volume at the onset of P wave (LAVp, r = 0.84, *P* < 0.001), and different stages of LV diastolic function. The LAVi increased as the severity of LV diastolic dysfunction increased.

**Conclusions:**

LA remodeling takes place in patients with LV diastolic dysfunction. At the same time, LA phasic volume and function parameters evaluated by DSCT indicated the severity of the LV diastolic dysfunction. Quantitative analysis of LA phasic volume and function parameters using DSCT could be a viable alternative prognostic parameter of LV diastolic function.

## Introduction

The left atrium serves as a reservoir, conduit, and booster pump during the cardiac cycle [1]. With its reservoir, conduit, and contractile functions, the left atrium plays an integral role in cardiac performance by modulating LV filling [2]. As the LV diastolic function deteriorates, the diastolic pressure in the left ventricle increases [3]. In order to preserve adequate LV filling pressure, the LA pressure and wall tension increase, and eventually the size of the left atrium increases [3]. It has been demonstrated that there is significant correlation between LAV and LV diastolic dysfunction [4]. However, almost all the results about LAV and LV diastolic dysfunction have been reported in studies involving echocardiography [5–9]. To date, there is no report about LAV and LV diastolic dysfunction evaluated by CT, and neither the relationship between LA phasic function and LV diastolic dysfunction has been evaluated by CT.

Coronary CT angiography (CCTA) examination has been widely used in clinical settings. Information regarding cardiac function can also be collected as a by-product of CCTA and can be used to evaluate heart function accurately [10–12]. In addition, LA phasic volume and function evaluation and information of coronary artery trees can be collected in one scan without additional radiation or contrast medium using CCTA examination. The advantage of DSCT coronary angiography is that it can reduce the dose exposure using DSCT ECG-gated tube current modulation technique for all the patients, as was previously recommended [13]. A previous study had demonstrated that it produces accurate and reproducible results in the assessment of LA volume and function using DSCT [10,14,15]. In most studies involving the CT evaluation of LA, the LA maximum volume, LA minimum volume and LAEF are usually evaluated, but LA phasic volume and function are seldom assessed by CT [16,17].

To date, there has been no report regarding the relationship of LA phasic volume and function with LV diastolic dysfunction using DSCT measurement. The purpose of this study was to assess the impact of LV diastolic dysfunction on LA phasic volume and function using DSCT and to find a viable alternative prognostic parameter of CT for LV diastolic dysfunction through quantitative evaluation of LA phasic volume and function in patients with LV diastolic dysfunction.

## Materials and Methods

### Ethics statement

The study protocols and written informed consent forms were approved by the local ethics committee of Beijing Anzhen Hospital associated with Capital Medical University in China. Written informed consent for information to be used for the study was obtained from all participating patients.

### Study population

Those patients who were referred for a clinically indicated CCTA were prospectively enrolled for possible participation in this study. The patients with arrhythmias, cardiac valvular heart diseases, congenital heart diseases, or implanted pacemakers or defibrillators were excluded. Additionally, patients were excluded if they could not undergo CCTA examination because of renal dysfunction (serum creatinine levels higher than 1.5 mg/dl) or a previous allergic reaction to iodinated contrast media. Only patients with normal LV systolic function were included (LVEF > 50%). A total of 77 patients (42 men and 35 women; mean age, 53.99 ± 12.90 years) met the study’s inclusion criteria. Of these, 49 had hypertension, 7 had history of myocardial infarction, 15 had coronary artery disease, and 26 had diabetes mellitus. For all the included patients, Doppler echocardiography was performed after CT on the same day. The subjects’ heart rates were continuously monitored throughout image acquisition by two modalities. No additional ß-blockers were administered in the current study.

### Echocardiographic imaging

A commercially available system, Philips IE33, was used for imaging. Pulsed wave (PW) Doppler and tissue Doppler (TD) were obtained using a S5-1 electronic transducer (1.7–3.4 MHz) and data were obtained from apical four-chamber view [18]. PW Doppler sample volume was placed parallel to the mitral valve flow to obtain Peak velocities of mitral inflows in early diastole (E), peak velocities of mitral inflows in atrial systole (A), and deceleration time of the E-wave velocity (DT). The E/A ratio was then calculated. The early diastolic mitral annular peak velocities of septal side (e′ s) and early diastolic mitral annular peak velocities of lateral side (e′ l) were measured at the level of the mitral annulus using tissue Doppler imaging. The average of e′ s and e′ l (em) and the average E/e′ of two side of mitral annulus (Av. E/e′) were then calculated. All echocardiographic parameters were measured online, and the average among 2 to 3 cardiac cycles was calculated.

### Classification of left ventricular diastolic function

The LV diastolic function was divided into four groups according to the recommendations for the Evaluation of Left Ventricular Diastolic Function by the American Society of Echocardiography and European Society of Echocardiography [19]. If e′ s ≥ 8 cm/s, e′ l ≥ 10 cm/s, E/A > 1, and 160 < DT < 200 ms, LV diastolic function was defined as normal, If e′ s < 8 cm/s, e′ l < 10 cm/s, and em < 8 cm/s, then LV diastolic function was divided into an impaired relaxation group (grade I) (E/A < 0.8, Av.E/e′ ≤ 8 cm/s, and DT > 200 ms); pseudonormal filling group (grade II) (0.8 < E/A < 1.5, 9 cm/s < Av.E/e′ ≤ 12 cm/s, and 160 < DT < 200 ms); restrictive filling group (grades III and IV) (E/A ≥ 2, Av.E/e′ ≥ 13 cm/s, and DT < 160 ms).

### DSCT imaging

CT examination was performed using a 64-slice DSCT scanner (Somatom Definition, Siemens Medical Solutions, Forchheim, Germany). The scanning range covered the entire heart from 1 cm below the level of the tracheal bifurcation to the diaphragm. A 50–75 ml bolus of Iopamidol 370 (Bracco, Shanghai, China) followed by 20 ml of saline solution was continuously injected into an antecubital vein at a rate of 4–5 ml/s via a dual-head power injector (Stellant; Medrad, Indianola, PA, U.S.). The CT angiography was triggered automatically and image acquisition started 4 s after the attenuation reached the predefined threshold of 100 HU at the aortic root. The data were acquired in a craniocaudal direction with a tube voltage of 100 kV—135 kV and tube current of 350 mAs—420 mAs. The other scan parameters were as follows: slice collimation of 2 × 32 × 0.6 mm, slice acquisition of 2 × 64 × 0.6 mm by means of a z-flying focal spot, detector collimation of 64 × 0.5 mm, a gantry rotation time of 330 ms, pitch of 0.2–0.5 (depending on the heart rate), reconstructed slice thickness of 0.75 mm (at 0.4 mm increments), and B26f kernels. Electrocardiography (ECG)-gated tube current modulation was used to reduce the required dose of radiation during scanning for all the patients, as recommended [13]. The estimated radiation dose in this study was about 6–10 mSv using this protocol. Retrospective ECG-gating was used to synchronize the data with the ECG, and 20 series of axial images were reconstructed every 5% of the R–R interval (0%–95%) with an effective slice thickness of 0.75 mm, a reconstruction increment of 0.4 mm and a field of view of 180 mm × 170 mm (matrix: 512 × 512). The images obtained at the onset of the P-wave on ECG were also reconstructed using the same parameters to obtain LAV before atrial active contraction. All the reconstructed DSCT data were post-processed on a dedicated workstation (Multi Modality Work Place, CT 2008A, Siemens Medical Solutions, Forchheim, Germany).

**Fig 1:**
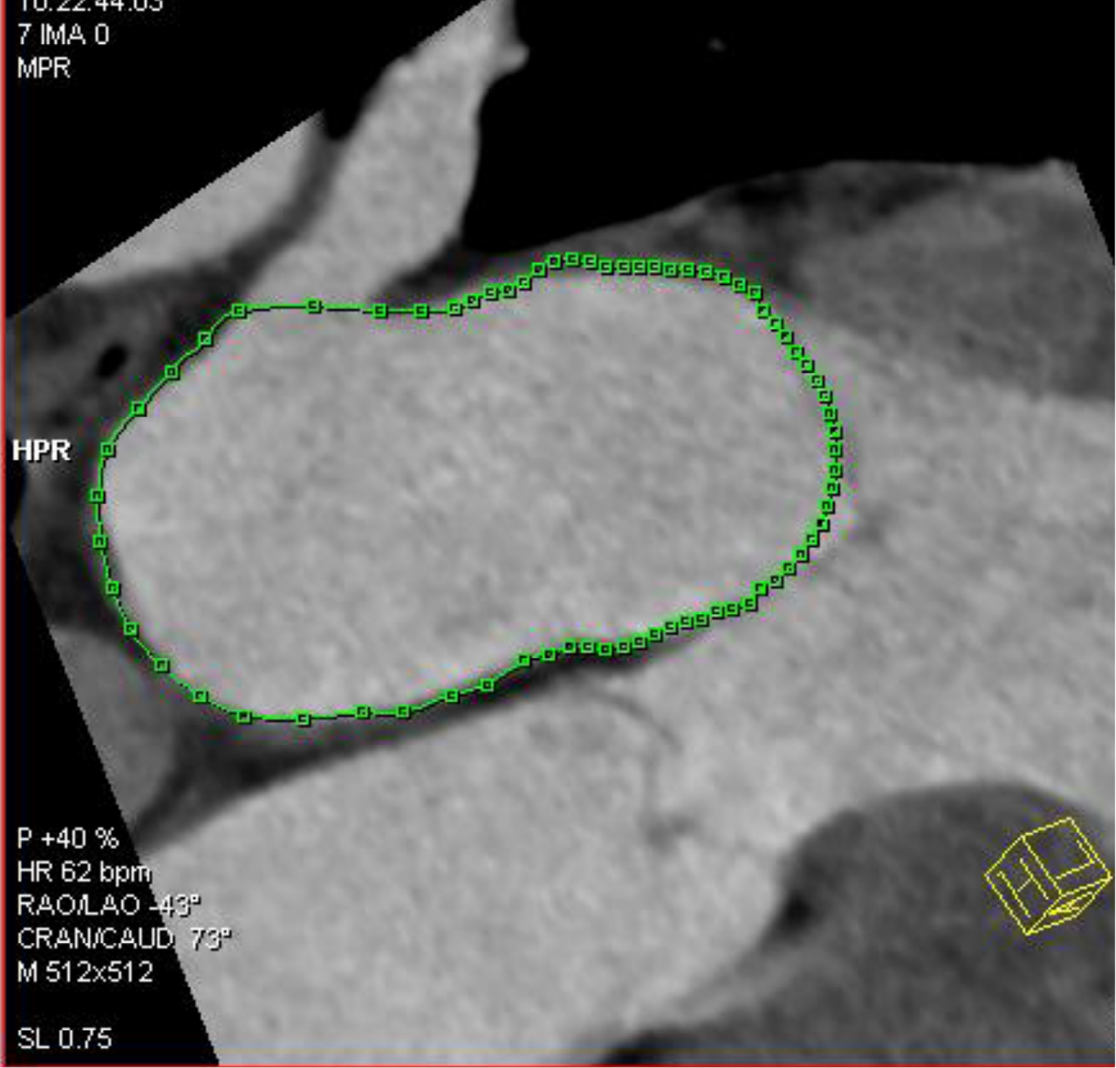
2D image of LA. Two-dimensional image illustrating how to delineate left atrial endocardium during postprocessing LA volume data.

**Fig 2:**
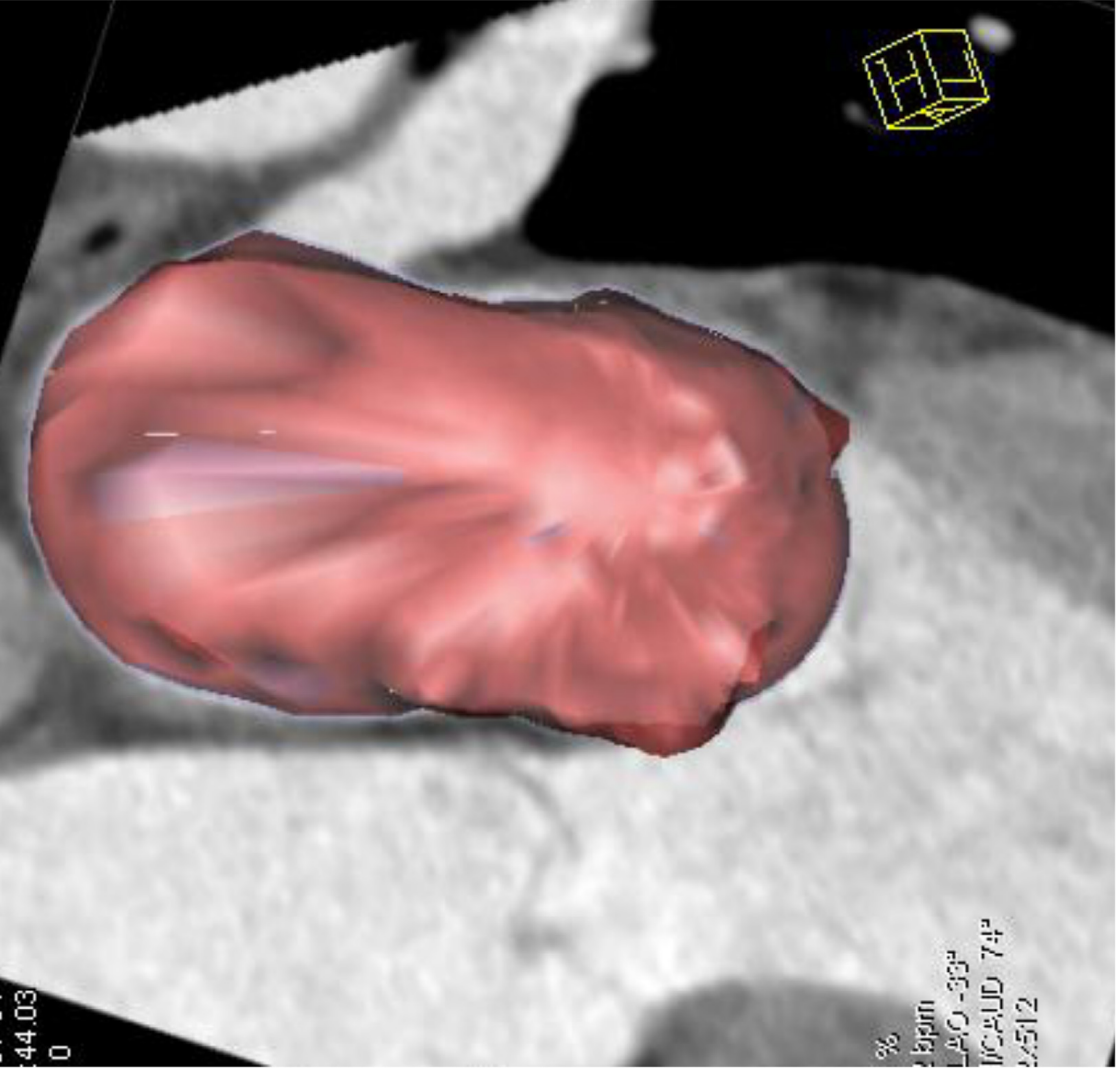
2D and 3D fusion image of LA. The fusion image providing true 3D rendering of LA without taking any geometrical assumptions.

Two independent observers with more than 5 years experience in cardiovascular imaging evaluated the images and measured the parameters of the left atrium. Assessment of the LAV was performed with a dedicated software package for cardiac function analysis (Circulation, Siemens Medical Solutions, Forchheim, Germany) with threshold-based region-growing 3D segmentation of the LA cavity. When necessary, the LA endocardial contours were manually corrected frame by frame on 360° rotational MPRs to make sure that all the CT images had a discernible cardiac cavity (Figs 1 and 2). The atrial appendages and pulmonary veins were excluded from the delineation of the LA endocardial border. The post-processing software automatically calculated the LA volumetric parameters. All the volumes were normalized to the body surface area (BSA).

### Index of LAV and function

The left atrial function was defined as follows [20]:

1. left atrial reservoir function = (LAVmax—LAVmin)/LAVmin × 100%
2. left atrial conduit function = (LAVmax—LAVp)/LAVmax × 100%
3. left atrial active contractile function = (LAVp—LAVmin)/ LAVp × 100%
4. LAEF = (LAVmax—LAVmin)/LAVmax × 100%.

All the volumes were normalized to the body surface area (BSA).

LAVmax = maximal left atrial volume

LAVmin = minimal left atrial volume

LAVp = left atrial volume at the onset of P wave

LAEF = left atrial ejection fraction.

### Statistical analysis

Results are presented as mean ± SD for continuous variables. The statistical software was SPSS version 18.0 (SPSS, Inc., Chicago, IL, U.S.). Differences in indexed LAV and function between groups with different levels of left ventricular diastolic dysfunction grade were analyzed using one-way analysis of variance followed by post-hoc test. The Spearman correlation coefficient was used to assess the correlation between the indexed LAV and different grades of LV diastolic function. For all statistical analyses, a *P* values below 0.05 was considered significant.

## Results

### 1. Clinical characteristics of different diastolic function groups

The clinical characteristics and echocardiographic variables are listed in Table 1. The E value were significantly lower in the impaired relaxation group (*P* < 0.001) and significantly higher in the restrictive filling group (*P* ≤ 0.01) than in the other 3 groups. On the contrary, the A value were significantly higher in the impaired relaxation group (*P* < 0.001) and significantly lower in the restrictive filling group (*P* ≤ 0.05) than in the other 3 groups. There were significant differences in E/A ratios between the normal group and LV diastolic dysfunction groups and with the other 3 groups (*P* < 0.01). In addition, e′ s, e′ l, and em were significantly lower in LV diastolic dysfunction groups than in the normal (*P* < 0.001). In the pseudonormal filling group and restrictive filling group, the Av. E/e values were more pronounced than in the normal group and impaired relaxation group (*P* < 0.001). DT time was significantly greater in the impaired relaxation group than in the other 3 groups (*P* < 0.001), but DT time was significantly lower in the restrictive filling group (*P* < 0.001). LVEF > 50% in all groups, but it was significantly lower in the pseudonormal filling group (*P* = 0.002) and restrictive filling group than in the normal group (*P* = 0.009).

**Table 1:** Clinical characteristics and echocardiographic variables of the normal group and LV diastolic dysfunction groups.

Characteristics	Normal	Impaired relaxation	Pseudonormal	Restrictive
	(n = 19)	(n = 26)	(n = 22)	(n = 10)
Male (%)	52.63	53.85	54.55	60.00
Age (year)	51.79±11.77	53.15±14.56	54.41±12.30	59.40±11.90
BMI	23.65±3.47	23.60±2.86	24.93±2.33	24.84±2.63
BSA (cm^2^)	1.76±0.19	1.80±0.19	1.80±0.16	1.80±0.26
E (cm/s)	80.78±17.64	50.89±9.33*	81.31±10.01	108.63±19.82*
A (cm/s)	57.48±15.91	93.30±17.18*	69.63±11.42	42.16±7.80*
E/A	1.44±0.26*	0.55±0.09*	1.19±0.19*	2.61±0.40*
e′ s (cm/s)	11.62±2.78*	6.17±1.25^§^	6.40±0.76^§^	4.60±0.97*
e′ l (cm/s)	13.83±2.89*	7.78±0.91^§^	8.15±0.96^§^	4.80±0.83*
em (cm/s)	12.73±2.73*	6.98±0.99^§^	7.28±0.73^§^	4.70±0.89*
Av. E/e′	6.51±1.40	7.43±0.64	11.36±0.69*	23.80±5.63*
DT (ms)	182.00±12.37	243.96±26.55*	181.73±11.86	128.90±12.86*
LVEF (%)	64.41±6.94	60.72±5.98	57.49±5.69^§^	56.74±3.99^§^
MI (%)	0.00	15.38	9.09	10.00
CAD (%)	0.00	23.08	36.36	10.00
DM (%)	26.32	30.77	40.91	40.00
HP (%)	15.79	76.92	86.36	70.00

MI: myocardial infarction; CAD: coronary artery disease; DM: diabetes mellitus; HP: systematic hypertension

*P < 0.05, relative to the other 3 groups,

^§^ *P* < 0.05, relative to the normal group

### 2. Differences in LA reservoir, conduit, and contractile function in different diastolic function groups measured by DSCT

The differences in LA reservoir, conduit, and contractile function in different diastolic function groups as measured by DSCT are given in Table 2. LA reservoir function was considerably lower in the pseudonormal group and restrictive filling group than in the impaired relaxation group and normal group (*P* < 0.001). The LA reservoir function in restrictive filling group was significantly lower than in the pseudonormal group (*P* < 0.001). There was no significant difference between impaired relaxation group and normal group for left atrial reservoir function (*P* = 0.181). There was also no significant difference between impaired relaxation group and normal group for left atrial conduit function (*P* = 1.000). The LA conduit function in pseudonormal group (*P* = 0.033) and in restrictive filling group (*P* < 0.001) was significantly lower than in normal group. The LA conduit function in restrictive filling group was significantly lower than in the impaired relaxation group (*P* = 0.001). There was no significant difference between the impaired relaxation group and normal group for LA contractile function (*P* = 1.000). The LA contractile function in the pseudonormal group and restrictive filling group was significantly lower than in the impaired relaxation group and normal group (*P* < 0.001). The LA contractile function in the restrictive filling group was significantly lower than in the pseudonormal group (*P* = 0.001). The indexed LAVmax, the indexed LAVmin, and the indexed LAVp in LV diastolic dysfunction groups were significantly higher than in the normal group (all *P* values < 0.05). The LA ejection fraction (LAEF) was lower in the impaired relaxation group than in the normal group but not significantly so (*P* = 0.139). The LAEF was significantly lower in the pseudonormal group and restrictive filling group than in the normal group and impaired relaxation group (all *P* values ≤ 0.001).

**Table 2:** Comparison of the LA volume and function among the 4 study groups.

LA volume and function	Normal	Impaired relaxation	Pseudonormal	Restrictive
	(n = 19)	(n = 26)	(n = 22)	(n = 10)
Indexed LAVmax (ml/m^2^)	23.32±5.10*	32.83±5.77*	40.83±5.21*	52.14±9.05*
Indexed LAVmin(ml/m^2^)	9.00±2.33*	13.62±2.79*	23.32±4.37*	37.73±9.48*
Indexed LAVp (ml/m^2^)	16.15±3.99*	23.39±4.20*	31.30±6.15*	43.49±10.95*
LAEF (%)	61.57±4.43	58.46±4.42	42.94±7.57*	28.23±7.88*
LA reservoir function	1.64±0.32	1.43±0.27	0.78±0.24*	0.41±0.17*
LA conduit function	0.31±0.07	0.28±0.07	0.24±0.09^§^	0.17±0.09^§^ ^&^
LA contractile function	0.44±0.07	0.41±0.08	0.25±0.09*	0.13±0.04*

*P < 0.05, Relative to the other 3 groups

^§^ *P* < 0.05, relative to the normal group,

^&^ *P* < 0.05, relative to the impaired relaxation group,

### 3. Correlation between indexed LAVmax, LAVmin, LAVp, and severity of LV diastolic dysfunction

**Fig 3:**
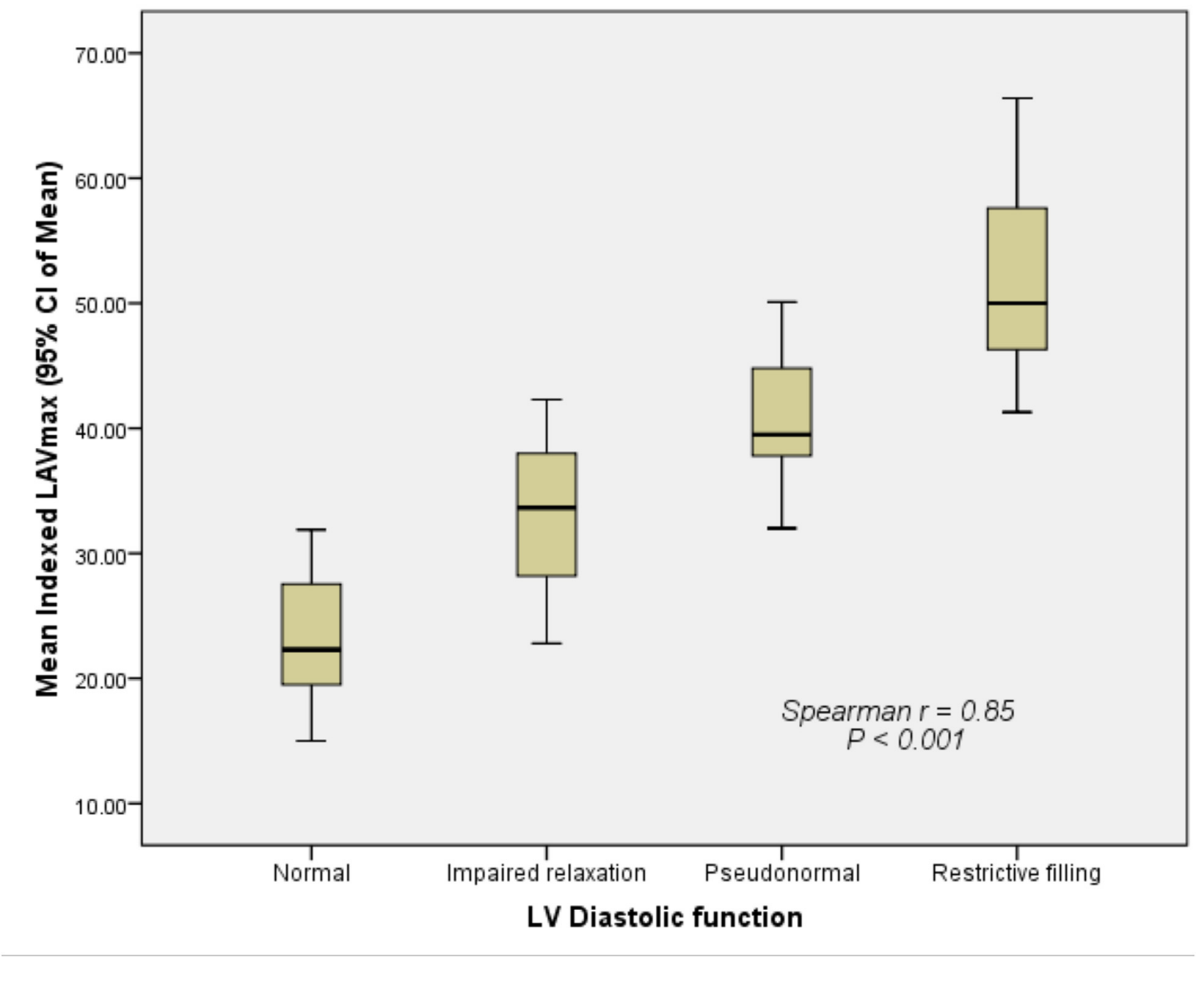
Correlation between the indexed LAVmax and different grades of left ventricular diastolic dysfunction. The indexed LAVmax increased significantly as LV diastolic dysfunction decreased (*P* < 0.001).

**Fig 4:**
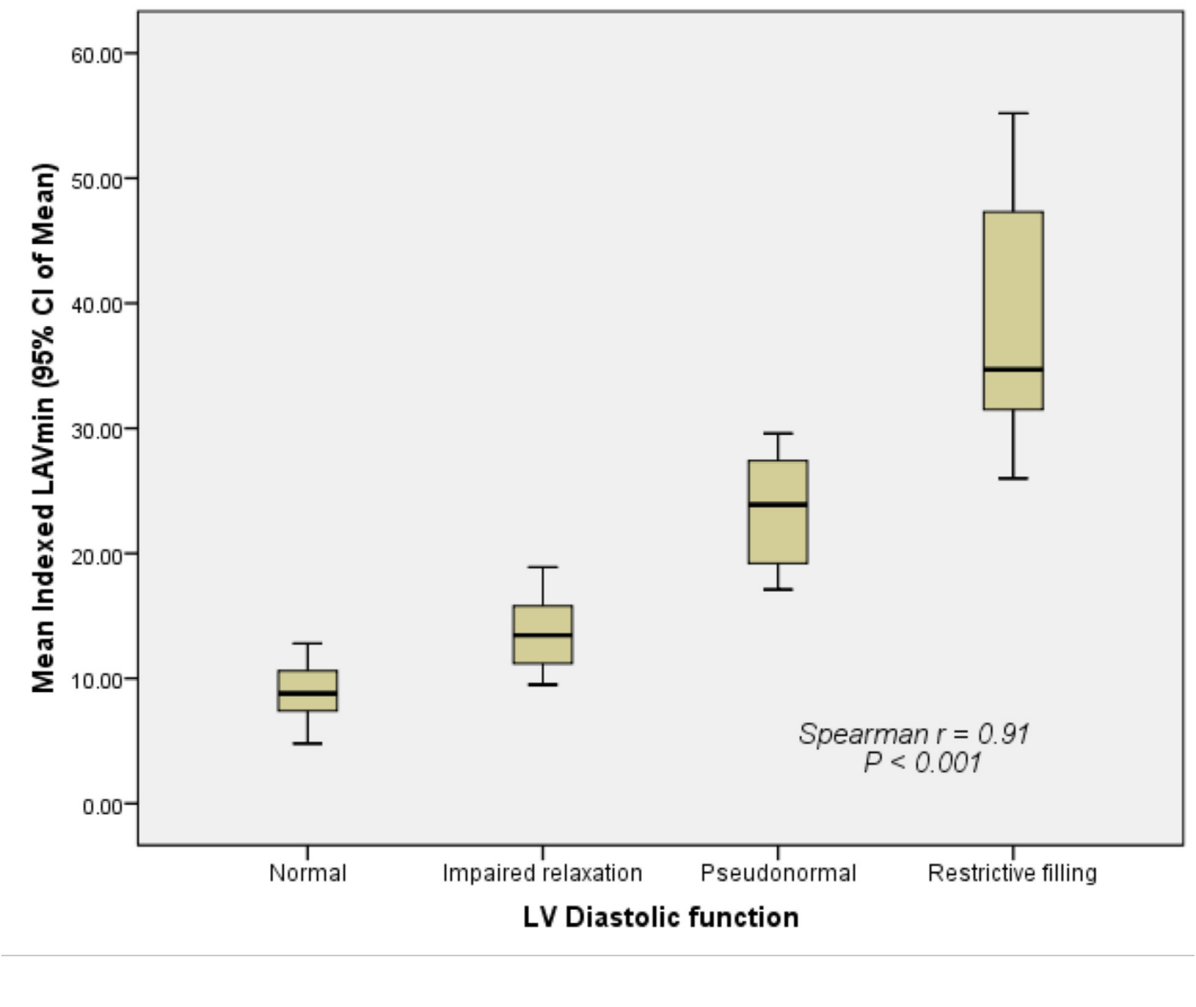
Correlation between the indexed LAVmin and different grades of left ventricular diastolic dysfunction. The indexed LAVmin increased significantly as LV diastolic dysfunction decreased (*P* < 0.001).

**Fig 5:**
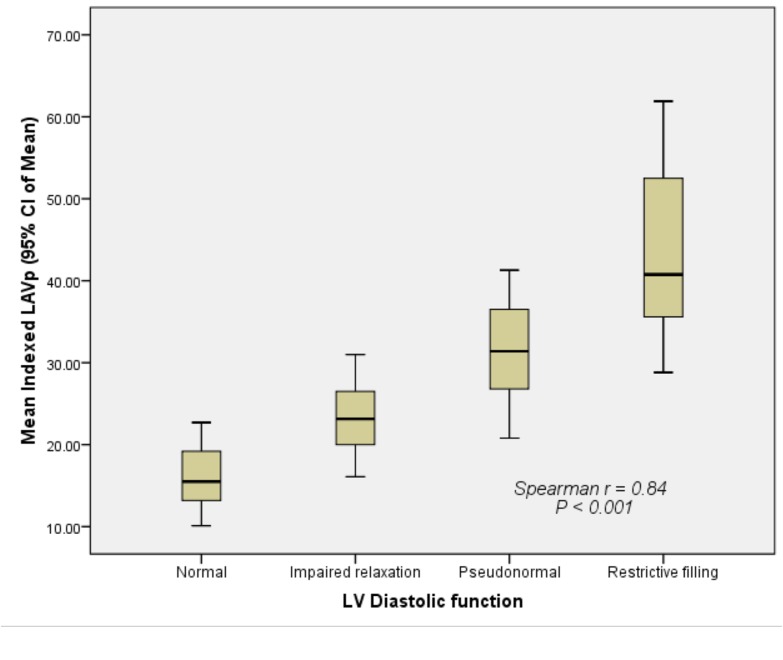
Correlation between the indexed LAVp and different grades of left ventricular diastolic dysfunction. The indexed LAVp increased significantly as LV diastolic dysfunction decreased (*P* < 0.001).

There was good correlation (r = 0.85) between the indexed LAVmax and different degrees of LV diastolic dysfunction (Fig 3). The indexed LAVmax increased significantly as LV diastolic dysfunction decreased (*P* < 0.001). Similarly, there was a strong correlation (r = 0.91) between the indexed LAVmin and different degrees of LV diastolic dysfunction (Fig 4). The indexed LAVmin increased significantly as the LV diastolic dysfunction decreased (*P* < 0.001). There was also a strong correlation (r = 0.84) between the indexed LAVp and different degrees of LV diastolic dysfunction (Fig 5). The indexed LAVp increased significantly as the LV diastolic dysfunction decreased (*P* < 0.001).

## Discussion

The main findings of the current study were that (a) left atrial reservoir, conduit, and contractile function decreases as the left ventricular diastolic function worsens, and (b) there is a significant correlation between indexed left atrial volume assessed by DSCT and different stages of LV diastolic dysfunction.

The current study demonstrated that there were no significant differences in LAEF between the impaired relaxation group and the normal group. There was also no significant difference in the LA contractile function, reservoir, or conduit function between these two groups. However, all these functions showed a downward trend. One explanation may be that there is a compensatory mechanism for the decreased LA functions that helps maintain total LA emptying volume [21]. However, in patients in pseudonormal group and restrictive group, LAEF, LA contractile function, and reservoir function deteriorated in parallel with the changes in LV diastolic dysfunction, becoming lower than in normal controls. This is because LA filling pressure increases as the severity of LV diastolic dysfunction increases [22]. The duration of increase in LA pressure becomes longer, and the LA wall tension increases, which causes LA dilatation and stretching of the LA myocardium. Eventually, LA mechanical dysfunction causes the loss of LA compensation, and LAEF, LA reservoir function, and contractile function decreased visibly [23]. There were no significant difference in LA conduit function between the patients in the pseudonormal group and restrictive filling group. This indicated that, at the end stage of LV diastolic dysfunction, LA mainly serves as a conduit. As in the current work, which involved two-dimensional volume indices and speckle tracking echocardiography, Santos et al. found that patients with heart failure with preserved ejection fraction (HFpEF) had worse LA reservoir, conduit, and pump function than controls [24]. In this way, LA phasic function evaluation by DSCT could be an index marker for identifying the LA compensatory stage and decompensated stage in patients with LV diastolic dysfunction.

There is a growing body of evidence demonstrating that LA enlargement is closely associated with the severity of diastolic dysfunction and can serve as predictor of future stroke, atrial fibrillation, and death [25–28]. In addition, the left atrium reflects left ventricular filling pressure and LA enlargement is a positive form of remodeling that occurs in response to increases in LV filling pressure. Tsang et al [29]. reported that LA volume provides a long-term view that may indicate the diastolic dysfunction of the patient regardless of the loading conditions of heart at the time of the examination and that LA size might be an important clinical risk stratified factor in preclinical cardiovascular disease.

Traditionally, echocardiography is first-line imaging modality for evaluation of LA size. However, in the patients with poor echo window, LA volume is impossible to evaluate. The evaluation of LA size and function by echocardiography is not accurate since using Simpson’s method, which involves making geometrical assumptions. And 3D echocardiographic quantitation of LA volume tends to skew results lower than magnetic resonance imaging (MRI) or CT techniques [30,31]. Cardiac MRI was found to accurately assess LA phasic volume and function during the heart cycle. However, it is usually impractical for routine clinical use with MRI because of its costs and limited availability. DSCT can be used to measure LA phasic volume and function reliably using real volume data acquisition and 3D segmentation. DSCT can also be used as an alternative means of evaluating LV diastolic dysfunction because it can provide cyclic changes in LA volume and function on the basis of true 3D rendering of the LA volume without geometrical assumptions [32]. For this reason, it is here suggested though LA phasic volume and function assessment, DSCT could reflect left ventricular diastolic function in different stages of LV diastolic dysfunction.

Previous studies involving echocardiography showed that indexed LAVmax has a strong graded correlation to the severity of LV diastolic function [23,33]. In this study, results showed that indexed LAVmax, indexed LAVmin, and indexed LAVp all had a strong graded correlation to the severity of LV diastolic dysfunction. The explanation is that during the cardiac cycle, LA is directly exposed to the increased LV filling pressure in LV diastolic dysfunction. In order to maintain LV filling, LA pressure increases, and eventually the LA wall stretches and LA increases in size. In this way, the LA phasic volumes may reflect LV filling pressure and may be capable of positive remodeling (dilating) in response to its elevation.

Although the feasibility of LA assessment by CT has been already reported [10,14], most studies about LA and CT reported only LAVmax, LAVmin and LAEF evaluation by CT. To date, there is no report about relationship between LAV and LV diastolic dysfunction evaluated by CT, and neither the relationship between LA phasic function (left atrial contractile function, conduit function and reservoir function) and LV diastolic dysfunction evaluated by CT. In addition, there are many modalities that could be used to evaluate LV diastolic dysfunction. Each modality has its own advantage and disadvantage for LV diastolic dysfunction measurement. LV diastolic function measurement by Doppler provdes information about a single point in time and may not reflect the severity of LV diastolic dysfunction over time, whereas increased LA size may reflect the cumulative effect of filling pressures over time. However, with real volume data acquisition and real three dimensional segmentation postprocessing technique, the use of DSCT, make the determinations of LA phasic volume and function more accurate and are therefore of value.

The current study has several limitations. First, the sample size was relatively small, because clinically, there are not as many patients with restrictive LV diastolic dysfunction as normal patients or patients with slight LV diastolic dysfunction. Second, Doppler echocardiography, not cardiac catheterization, was used to evaluate the left ventricular diastolic function for the classification of LV diastolic function grades. However, its invasive nature renders it impractical as a clinical tool. Future studies will have to be performed to demonstrate the prognostic value of LA phasic volume and function and facilitate the development of more effective strategies for the treatment and prevention of LV diastolic dysfunction.

## Conclusions

In summary, LA remodeling occurs in patients with LV diastolic dysfunction. LA phasic volume and function parameters evaluated by DSCT were found to indicate the severity of the LV diastolic dysfunction. Quantitative analysis of LA phasic volume and function parameters using DSCT was found to be a viable alternative prognostic parameter for LV diastolic dysfunction.

## Supporting Information

S1 Dataset Clinical characteristics and echocardiographic measurements of the 4 study groups. (XLS)

S2 Dataset LA volume and function measurements of the 4 study groups. (XLS)
